# Exploring the tumor-suppressive role of miRNA-200c in head and neck squamous cell carcinoma: Potential and mechanisms of exosome-mediated delivery for therapeutic applications

**DOI:** 10.1016/j.tranon.2024.102216

**Published:** 2024-11-29

**Authors:** Mohamed S. Kishta, Aya Khamis, Hafez AM, Abdelrahman H. Elshaar, Désirée Gül

**Affiliations:** a Hormones Department, Medical Research and Clinical Studies Institute, Stem Cell Lab., Center of Excellence for Advanced Sciences, National Research Centre, 33 El Bohouth St., Dokki, 12622 Cairo, Egypt; b Maxillofacial and Oral Surgery, University Medical Center, 55131 Mainz, Germany; c Oral Pathology Department, Faculty of Dentistry, Alexandria University, 5372066 Alexandria, Egypt; d Medical Biochemistry Department Faculty of medicine KafrElSheikh University, Kafr El-Sheikh, Egypt; e Faculty of Oral and Dental Medicine, Future University, Cairo, Egypt; f Department of Otorhinolaryngology Head and Neck Surgery, Molecular and Cellular Oncology, University Medical Center, 55131 Mainz, Germany

**Keywords:** Head and neck squamous cell carcinoma, microRNA, Epithelial-mesenchymal transition, Exosomes, Targeted therapy

## Abstract

Head and neck squamous cell carcinoma (HNSCC) remains a challenging malignancy due to its high rates of recurrence, metastasis, and resistance to conventional therapies. microRNA-200c (miRNA-200c) has emerged as a critical tumor suppressor in HNSCC, with the potential to inhibit epithelial-mesenchymal transition (EMT), which is considered as a key process in cancer metastasis and progression. Interestingly, there are also controversial findings in HNSCC characterizing miRNA-200c as oncogenic factor. This review article provides a comprehensive overview of the current understanding of miRNA-200c's general role in cancer, and particularly in HNSCC, highlighting its mechanisms of action, including the regulation of EMT and other oncogenic pathways.

Additionally, the review explores the innovative approach of exosome-mediated delivery of miRNA-200c as a therapeutic strategy. Exosomes, as natural nanocarriers, offer a promising vehicle for the targeted delivery of miRNA-200c to tumor cells, potentially overcoming the limitations of traditional delivery methods and enhancing therapeutic efficacy. The review also discusses the challenges and future directions in the clinical application of miRNA-200c, particularly focusing on its potential to improve outcomes for HNSCC patients. This article seeks to provide valuable insights for researchers and clinicians working towards innovative treatments for this aggressive cancer type.

## Highlights

• Review examines miRNA-200c's roles in oncogenic pathways.
• Analysis of miRNA-200c´s dual role in head and neck cancer (HNSCC).
• Exosome-mediated delivery of miRNA-200c is discussed as an innovative therapeutic strategy.
• Challenges in clinical application of miRNA-200c are elucidated.

## Graphical abstract

**Figure:**
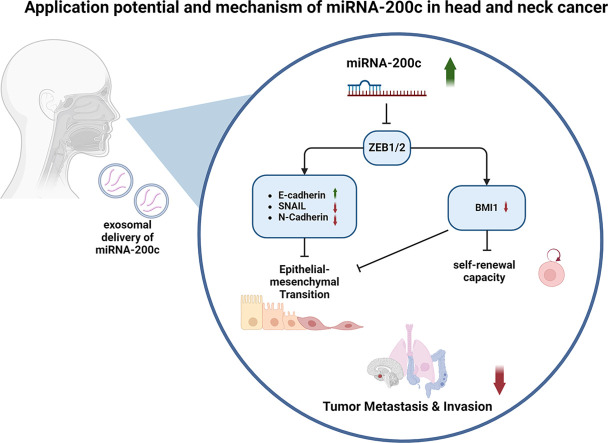

## Introduction

microRNAs (miRNAs) are small non-coding RNA molecules that regulate gene expression by binding to mRNA, typically at the 3′ untranslated region, either inhibiting protein translation or initiating mRNA degradation. They are crucial in various biological functions such as cell proliferation, differentiation, apoptosis, migration, and drug resistance. Aberrant miRNA expression can contribute to cancer progression or suppression [1]. Specifically, miRNA-200c-3p has been widely studied for its role in inhibiting epithelial-mesenchymal transition (EMT), thereby suppressing tumor invasion and metastasis. Downregulation of miRNA-200c is linked to increased invasion and metastasis in several cancers, including head and neck squamous cell carcinoma (HNSCC) [2].

HNSCC, the sixth most common cancer globally, is associated with risk factors like tobacco use, alcohol consumption, and HPV infection [3]. Despite advancements in treatment, the prognosis for HNSCC remains poor due to late-stage diagnosis, high recurrence rates, and resistance to conventional therapies, with a 5-year survival rate of around 50 %. This highlights the need for innovative treatments [4].

Exosomes, small extracellular vesicles, are gaining attention as potential delivery vehicles for cancer therapies due to their ability to transport bioactive molecules between cells, biocompatibility, and low immunogenicity. They can be engineered to carry therapeutic agents, such as miRNAs, directly to tumor cells, offering a promising approach for treating HNSCC by targeting metastatic recurrence and minimizing chemotherapy-related cytotoxicity [5].

In the following sections, we are providing a comprehensive overview of miRNA biology in general, as well as of the miRNA-200 family in particular including their role in EMT and cancer. Importantly, we discuss also the controversial findings in HNSCC where miRNA-200c can also execute oncogenic functions increasing therapy resistance. It also explores the potential of exosomes as nanocarriers for the therapeutic delivery of miRNA-200c, addressing the challenges and future directions in its clinical application for HNSCC therapy.

## Biogenesis of miRNA

**Fig. 1:**
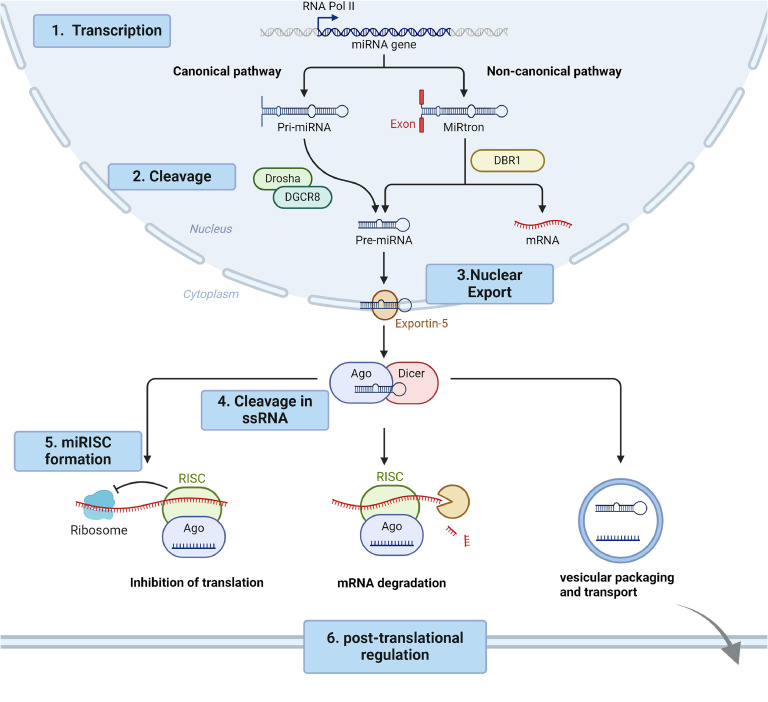
Schematic overview of the microRNA (miRNA) biogenesis. For details, see text. Created with BioRender.com.

The biogenesis of miRNA is a multi-step process that involves the transcription, processing, and maturation of miRNA molecules. Most microRNAs are generated via the canonical pathway, although a subset is generated through non-canonical pathways (Fig. 1).

### Canonical pathway

miRNA genes are transcribed by RNA polymerase II (and sometimes RNA polymerase III) into primary miRNAs (pri-miRNAs). These pri-miRNAs are typically several kilobases long and contain one or more stem-loop structures [6]. Subsequently, microprocessors like the RNase III enzyme Drosha and its cofactor DGCR8 (DiGeorge Syndrome Critical Region 8) cleave the pri-miRNA in the nucleus, producing a precursor miRNA (pre-miRNA) that is about 70 nucleotides long and has a characteristic hairpin structure [7] (Fig. 1). With the aid of the nuclear protein Exportin 5, the pre-miRNA is exported to cytosol where it undergoes processing by another RNase III enzyme, namely Dicer, leading to the formation of a mature miRNA duplex [8].

Dicer plays a crucial role that help miRNAs binding to mRNA for gene silencing. It cleaves the miRNA duplex into two strands, the guide and the passenger strand. The guide strand associates with Argonaute (AGO) to form the miRNA-induced silencing complex (miRISC), which facilitates the binding of miRISC to the target mRNA sequence. The passenger strand is subsequently degraded. Depending on whether the resulting miRNA originate from the 5′ or the 3′ end of the pre-miRNA, the miRNA is designated with either '−5p' or '−3p’, respectively [9].

### Non-canonical pathways

Some miRNAs are produced through non-canonical pathways. For instance, pri-miRNAs transcribed from miRtrons, which are situated within the intronic regions of protein-coding genes, cannot be cleaved by Drosha and DGCR8 (Fig. 1, right). Instead, they undergo splicing by debranching enzyme 1 (DBR1), resulting in a shorter sequence [10].

## The miRNA-200 family – ‘stars in the microRNA universe’

**Fig. 2:**
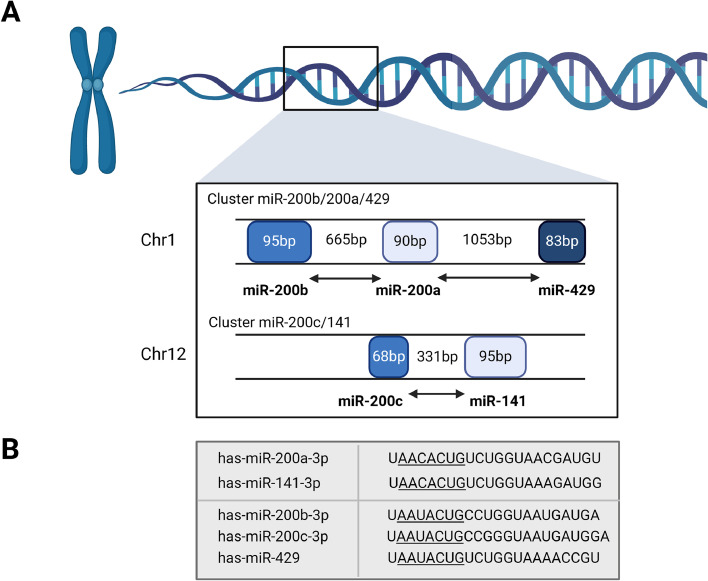
Chromosomal organization and sequence of the miRNA-200 family. A. The miRNA-200 family can be divided into two clusters according to the location on the chromosome. The first cluster located on chromosome 1 contains miRNA-200b, miRNA-200a and miRNA-429 (upper panel). The second cluster located on chromosome 12 consists of miRNA-200c and miRNA-141 (lower panel). B. The sequences of the mature miRNA-200 family members. The miRNA-200 family members can also be separated into two functional groups based upon their seed sequences (underlined). Functional group I is composed of miRNA-200b, −200c, and −429 and functional group II consists of miR-141 and-200a. The seed sequences of these two functional groups only differs by one nucleotide. All miRNA sequences shown here can be found in human which is indicated by ‘has’ in miRNA name. Created with BioRender.com.

The miRNA-200 family has gained high attention among cancer researchers due to their crucial roles in EMT regulation, and thus tumor metastasis, and modulation of chemoresistance. The miRNA-200 family consists of five miRNA members: miRNA-200a, miRNA-200b, miRNA-200c, miRNA-141, and miR-429. These miRNAs are clustered and expressed as two distinct polycistronic pri-miRNA transcripts, namely miRNA-200a, miRNA-200b, and miRNA-429 (located on chromosome 1), as well as miRNA-200c and miRNA-141 (located on chromosome 12) (Fig. 2A) [11]. The classification of the miRNA-200 family members into these two groups is based on their seed sequence differing in the third nucleotide (Fig. 2B). The mature −3p miRNA products derived from these precursors are prevalent in epithelial cells. Here, the miRNAs preserve the epithelial phenotype by suppressing EMT-promoting factors, a pivotal characteristic in oncogenic transformation [12].

### The role of miRNA-200 in epithelial mesenchymal transition (EMT)

Normal epithelial cells maintain cell polarity and tight adhesion through specific proteins, but the activation of EMT-inducing transcription factors (EMT-TFs) leads to the repression of these epithelial genes, causing a loss of cell-cell junctions and polarity. This transition allows cells to adopt a mesenchymal phenotype with increased motility, facilitating tumor cell invasion and metastasis [2]. The concept of EMT, first suggested in 1982, includes three primary types: Type I (embryogenesis), Type II (wound healing, tissue regeneration, and fibrosis), and Type III (cancer metastasis) [13]. EMT is marked by a decrease in cell-cell adhesion proteins like E-cadherin and an increase in mesenchymal markers like vimentin. This process not only promotes metastasis, but also contributes to chemoresistance and reduced apoptosis sensitivity in cancer cells [14]. The miRNA-200 family plays a tumor-suppressive role by maintaining the epithelial phenotype and inhibiting the mesenchymal transition [2], which is discussed in more detail in the following chapters.

#### miRNA-200c and the transcription factors ZEB

The miRNA-200 family targets the transcription factors ZEB1 and ZEB2, which are key regulators of epithelial-mesenchymal transition (EMT) [2]. The ZEB (Zinc finger E-box binding homeobox) family consists of two key members, ZEB1 and ZEB2 which repress the expression of E-cadherin, leading to the loss of epithelial characteristics and promoting tumor invasiveness. ZEB1, which can bind to nearly 2000 genes, is associated with increased cancer cell migration, survival, and invasion. Factors like the tumor suppressor DAXX and ROCK2 in pancreatic cancer can modulate ZEB1′s effects, influencing cancer progression and drug resistance. Similarly, ZEB2 promotes migration and invasion in non-small cell lung cancer by upregulating EMT-related proteins like MMP-9 and Twist [15].

The overexpression of miRNA-200 inhibits EMT by suppressing ZEB1 and ZEB2, maintaining E-cadherin expression and preventing metastasis. ZEB1/2 and miRNA-200 exhibit a dual-negative feedback loop, where they mutually suppress each other's expression (for details please refer to other reviews, e.g. [15]). Additionally, p53 can downregulate ZEB1/2 by activating miRNA-200 and miRNA-192, further inhibiting EMT. A TGF-β/ZEB/miRNA-200 signaling network has been identified, regulating the balance between epithelial and mesenchymal states in cancer cells. This mechanism was supported by the findings of Harb et al. who found high ZEB1 expression in the invasive area of malignant cells accompanied by decreased membranous expression of E-cadherin facilitating tumor invasiveness [16].

#### miRNA-200c and TGF-β regulate EMT

The TGF-β superfamily encompasses different subgroups of structurally related proteins, namely the TGF-β subfamily with three isoforms, the activin and inhibin subfamilies, bone morphogenetic proteins (BMPs), as well as other growth and differentiation factors [17]. They govern various cellular processes such as cell growth, differentiation, adhesion, migration, and apoptosis, exhibiting specificity depending on the cellular context and type [18].

TGF-β signaling plays pivotal roles in cancer progression via several key processes. First, TGF-β signaling modulates cellular growth, particularly by evading growth inhibition during early stages of cancer initiation. Secondly, activation of TGF-β can lead to an increased synthesis of extracellular matrix and induction of fibrosis within the tumor microenvironment. Lastly, promotion of EMT and/or metastasis, as well as immune suppression in the tumor microenvironment was observed after TGF-β activation [19].

**Fig. 3:**
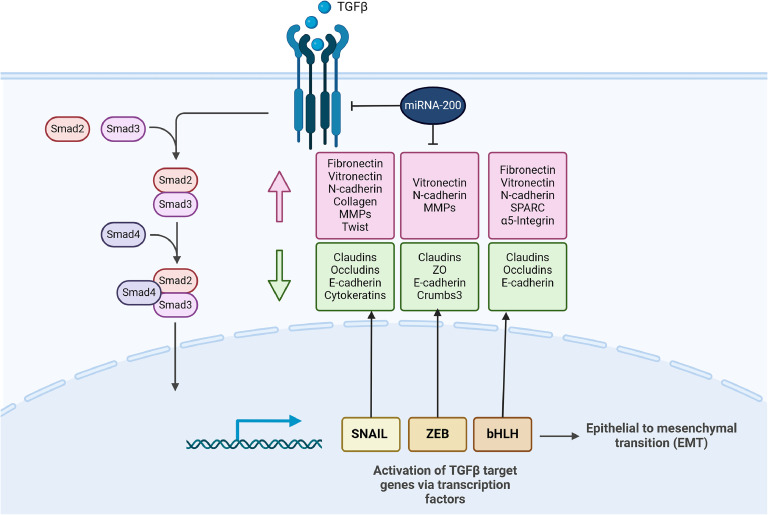
Transcriptional regulation of epithelial-mesenchymal transition (EMT) by TGF-β and interplay with miRNA-200. In response to TGF-β, Smad2 and 3 are activated, and form complexes with Smad4, which then regulate transcription of target genes through interactions with other DNA binding transcription factors. In the induction of EMT, the activated Smads mediate transcriptional regulation through three families of transcription factors (SNAIL, ZEB, bHLH), resulting in repression of epithelial marker gene expression (green boxes) and activation of mesenchymal gene expression (red boxes), and thus EMT. Increased expression of miRNA-200 family members is not only able to inhibit TGF-β-induced EMT, but can also induce the reverse effect, mesenchymal-epithelial transition (MET). Created with BioRender.com. (For interpretation of the references to color in this figure legend, the reader is referred to the web version of this article.)

TGF-β is a major inducer of EMT (Fig. 3). It interacts with its specific receptors (TGF-β RI-III), initiating phosphorylation of Smad 2 and Smad 3. These molecules subsequently form trimers with Smad 4, which then translocate into the nucleus. Inside the nucleus, this complex upregulates expression of transcription factors, e.g., ZEB, resulting in subsequent enhancement of EMT [20] (Fig. 3).

Furthermore, TGF-β signaling has been suggested to influence miRNA transcriptional and post-transcriptional regulation. The members of the miRNA-200 family are downregulated by TGF-β, specifically by TGF-β receptor type I (TβR-I), and Smad2. This TGF-β-mediated reduction of miRNA-200 expression additionally leads to enhanced TGF-β signaling and promotes EMT [21].

In agreement with previous data, it was reported that TGF-β treatment of different cell lines resulted in significant downregulation of miRNA-200 family members [22]. Here, the promoter regions of miRNA-200c are inhibited by the transcriptional repressors ZEB1 and ZEB2 induced by TGF-β. Interestingly, enforced expression of miRNA-200 s effectively prevented TGF-β-induced EMT and promoted the reverse mechanism, namely mesenchymal-epithelial transition (MET) in mesenchymal cells [23] (Fig. 3).

### miRNA-200c –dependent regulation of AKT/PI3K signaling in cancer

Many cancer cells exhibit elevated levels of AKT, a key regulator of various cellular processes, including apoptosis, autophagy, endoplasmic reticulum (ER) stress, and ferroptosis [24].

**Fig. 4:**
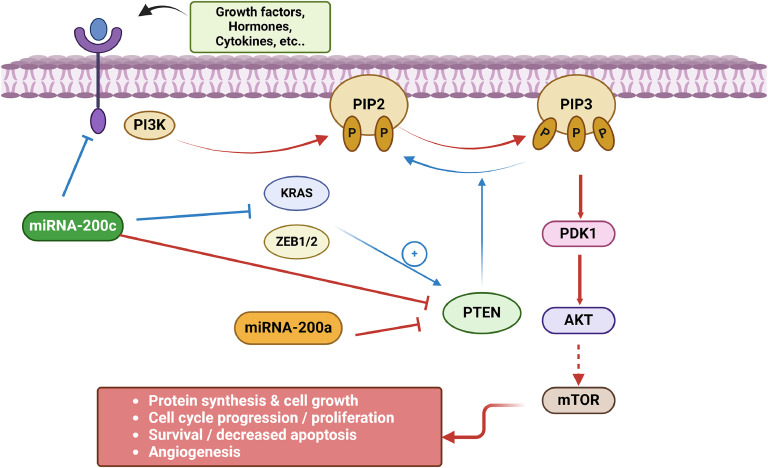
The miRNA-200 family regulates PI3K/AKT/mTOR signaling in cancer. miRNA-200 can execute dual functions in cancer leading to activation (red lines) or inactivation (blue lines) of PI3K/AKT signaling. miRNA-200c can suppress AKT signaling in tumor cells by its inhibitory effect on PI3K and other cell cycle modulators, such as ZEB1/2 and KRAS resulting in induction of the tumor suppressor PTEN. In some cancers, direct targeting of PTEN by miRNA-200c may reduce PTEN levels, promoting oncogenic signaling and aiding tumor growth. miRNA-200a has an additional inhibitory role on PTEN leading to cell proliferation. Red lines: oncogenic functions resulting in increased proliferation and survival (red box); blue lines: tumor-suppressive functions reducing PI3K/AKT signaling. Created with BioRender.com. (For interpretation of the references to color in this figure legend, the reader is referred to the web version of this article.)

Many studies have highlighted the role of miRNAs in modulating AKT signaling across various cancer types [25], including breast cancer [26], glioblastoma [27], and nasopharyngeal carcinoma [28]. Downregulation of AKT1/2 by miRNAs inhibits cancer cell proliferation in vitro and tumor growth in vivo. Additionally, the miR-200 family targets PTEN, a crucial inhibitor of the PI3K/AKT pathway. Specifically, miR-200a targets PTEN in endometrial cancer and esophageal carcinoma [29,30]. Notably, miRNA-200c has been shown to interact with KRAS [28] and inhibit PI3K/AKT signaling in breast cancer by upregulating PTEN (Phosphatase and Tensin Homolog) [26,31]. Specifically, miRNA-200c targets PIK3CB, a catalytic subunit of PI3K, thereby reducing the activity of the pathway [32] (Fig. 4).

Conversely, in nasopharyngeal carcinoma, miRNA-200c exhibits an oncogenic role, which is at least partially due to its suppression of PTEN by targeting the PTEN 3′ UTR [28,33]. Additionally, inhibition of miRNA-200c results in reduced cell viability, cell cycle arrest in the G0–G1 phase, and diminished cell migration and invasiveness [34].

### miRNA-200c regulate apoptosis in cancer

miRNA-200c also has a pro-apoptotic effect in all studied types of cancer, including breast, colorectal, gastric, and liver cancer, as well as NSCLC, and nephroblastoma [25,35]. For example, in triple-negative breast cancer cells, microRNA-200c was found to downregulate XIAP expression and thus promote apoptosis [36]. XIAP (X-linked inhibitor of apoptosis protein) plays a significant role in regulating apoptosis by directly inhibiting caspases-3, −7, and −9 [37].

Furthermore, in gastric cancer, miRNA-200c promotes apoptosis of the tumor cells by downregulation of endothelin receptor A (EDNRA) expression. Endothelin is a peptide that acts as a potent vasoconstrictor and is involved in various physiological processes, including cell proliferation, differentiation, and apoptosis. Endothelin binds to and activates two main receptor subtypes: endothelin receptor A (EDNRA) and endothelin receptor B (EDNRB). EDNRA is particularly associated with signaling pathways that regulate cell survival and apoptosis [25,38].

Similarly, in human hepatocellular carcinoma miRNA-200c induces apoptosis via suppressing MAD2L1 (Mitotic spindle assembly checkpoint protein, MAD2A) [39]. Suppression of MAD2L1 can lead to improper chromosome segregation, resulting in genomic instability and induction of intrinsic apoptotic pathways [40].

Interestingly, induction of miRNA-200 expression has been suggested as therapeutic intervention for cancer to trigger apoptosis and enhance responsiveness to chemotherapeutic drugs. For example, niclosamide (an antihelminth used against tapeworm infections) induced apoptosis in colon cancer cells by increasing the levels of miRNA-200 family members [41].

### miRNA-200c as immunomodulator in cancer

**Fig. 5:**
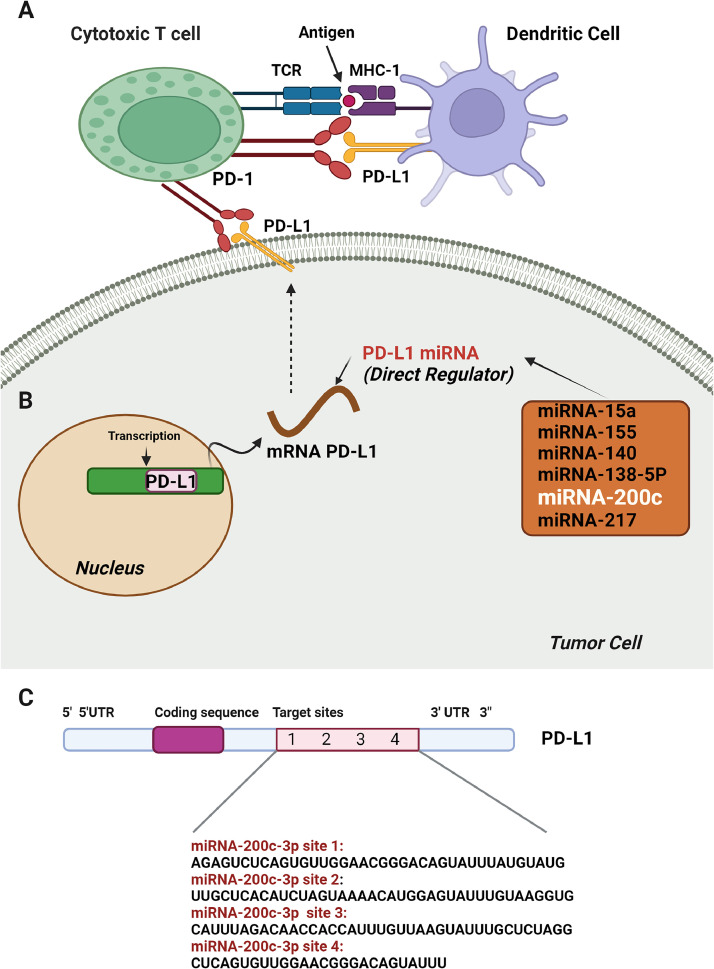
Role of miRNAs in PD-1/PD-L1-dependent immunomodulation. A. Schematic illustration of the PD-1/PD-L1 interaction resulting in T-cell exhaustion. B. Modulation of PD-L1 expression by miRNAs. Shown miRNAs are able to downregulate PD-L1 expression by targeting the 3′-UTR of PD-L1 mRNA. miRNA-200c is one of the direct regulators that can modulate the PD-1/PD-L1 axis by the shown mechanism. C. Schematic view and sequences of predicted binding sites for miRNA-200c-3p on human PD-L1 mRNA. (MHC-1, major histocompatibility complex type 1; PD-1, programmed cell death protein-1; PD-L1, programmed death-ligand 1; TCR, T cell receptor; 3′/5′UTR, 3′/5′ untranslated region) Created with BioRender.com.

Immune cells are designed to identify and eliminate "foreign" cancer cells, but cancer cells can evade immune surveillance through various mechanisms. One of the relevant key strategies is the immune checkpoint pathway [42]. A crucial immune checkpoint receptor is PD-1 (programmed cell death protein 1) which is present on antigen-specific T cells, and interacts with its ligand, the programmed cell death ligand 1 (PD-L1). Within the tumor microenvironment (TME), PD-1/PD-L1 binding can transmit regulatory signals to effector T cells, leading to T cell exhaustion. Simultaneously, this interaction provides anti-apoptotic signals to the tumor cells, aiding their survival and significantly dampening the immune response [43,44] (Fig. 5).

Interestingly, miRNA-200c was shown to influence the expression of PD-L1 via three or four binding sites identified on the PD-L1 mRNA in both mice and human respectively (Fig. 5C) [45]. It was shown before that treatment with miRNA-200c reduced PD-L1 expression in lung tumor cells [46]. Additionally, miRNA-200c can lower the number of T-regulatory cells, increase CD4+ and CD8+ *T* cells within tumors, and enhance the proliferation and effector functions of tumor-specific CD8+ *T* cells by targeting the PD-1/PD-L1 immune checkpoint pathway [47]. Furthermore, miRNA-200c modulates PD-L1 mRNA stability post-transcriptionally, and PD-L1 expression correlates negatively with miRNA-200c expression [48]. While in a study on osteosarcoma, MicroRNA sequencing revealed that microRNA-200a regulates PD-L1 expression in osteosarcoma cell lines U2OS, 143B, and K7. Overexpression of microRNA-200a led to an increase in PD-L1 levels in these cells. Also, the researchers concluded that PTEN as a direct target of microRNA-200a, is contributing to the upregulation of PD-L1. microRNA-200a promoted tumor growth by increasing Foxp3+ regulatory T cells while decreasing the proportions of CD4+, CD8+, and IFN-γ+ cytotoxic T cells. Interestingly, tumors overexpressing microRNA-200a responded more effectively to PD-L1-targeted immunotherapy [49].

Conversely, it has been reported that miRNA-200c can enhance the immunosuppressive function of myeloid-derived suppressor cells (MDSCs). These cells play a significant role in shielding cancer cells from detection and elimination by the immune system [50].MDSCs are a group of immunosuppressive cells, which differentiate from myeloid cells stimulated by chronic inflammation and other pathological conditions [51].

MDSCs can inhibit immune responses by producing reactive oxygen species (ROS), hydrogen peroxide (H_2_O_2_), nitric oxide (NO), and arginase [52]. Two key signaling pathways regulate the differentiation and suppressive functions of MDSCs [51]. One pathway drives MDSC expansion, while the other controls their activation [53]. Tumor-derived cytokines such as GM-CSF, M-CSF, G-CSF, IL-6, and VEGF trigger the expansion pathway, leading to STAT3 and STAT5 activation [50]. This disrupts normal myeloid development and promotes the proliferation of immature myeloid cells. STAT3, in particular, upregulates anti-apoptotic and pro-proliferative factors like BCL-XL, survivin, cyclin D1, and c-myc, enhancing myeloid cell expansion. It also increases the expression of pro-inflammatory proteins such as S100A8, S100A9, and Nox2, which contribute to MDSC differentiation and suppression [53].

Activation of MDSCs is driven by pro-inflammatory signals, including IFNγ, IL-1β, IL-4, PGE2, and LPS, which activate signaling pathways such as STAT1, STAT6, PI3K/AKT, and NF-κB. The PI3K/AKT pathway is particularly important for both MDSC activation and tumor-associated MDSC expansion [54,55]. miRNA-200c significantly enhances the suppressive capabilities of MDSCs. This occurs through targeting and downregulation of the proteins PTEN and FOG2 [56]. By binding to a regulatory subunit of PI3K, FOG2 also acts as a negative modulator of the PI3K/AKT pathway [57]. Consequently, downregulation of PTEN and FOG2 by miRNA-200c leads to the activation of the STAT3 and PI3K/AKT signaling pathways within MDSCs. Importantly, the cytokine GM-CSF has been identified as a key factor that induces the expression of miRNA-200c in the tumor microenvironment. In other words, the upregulation of miRNA-200c, driven by GM-CSF in the tumor setting, boosts the immunosuppressive functions of MDSCs [58].

### miRNA-200c in HNSCC

Head and neck squamous cell carcinoma (HNSCC) represent one of the most prevalent and lethal cancers globally. The primary challenges in improving patient outcomes are its late detection and the high heterogeneity of the tumors, which contribute to the suboptimal efficacy of current therapies. Since novel treatment options are urgently needed for HNSCC patients, the use of miRNAs can be a promising approach to increase patient outcome [2]. miRNA-200c represents a promising therapeutic agent due to its previously mentioned roles in tumor suppression, metastasis, and therapy resistance. Importantly, various studies demonstrated that miRNA-200 family members are downregulated in HNSCC and are involved in the EMT process of HNSCC tumorigenesis [59,60]. In particular, miRNA-200c was identified as negative regulator of BMI1 expression, and thus significantly inhibited EMT in malignant HNSCC [61]. The authors of the relevant study showed that the enforced expression of miRNA-200c significantly inhibited migration of HNSCC cells characterized by increased E-cadherin and decreased ZEB1 expression [62,63]. QKI is an important regulator of EMT that has been reported to increase during EMT and modulate EMT-related phenotypes. Previous studies have reported QK1 as a target for miRNA-200 family members [64].

Interestingly, there exist also controversial findings about the role of miRNA-200c in HNSCC. A recent study identified miRNA-200c as an oncogenic factor in esophageal squamous cell carcinoma (ESCC), where it can function as an oncomir [65]. Oncomirs can play a dual role in cancer by either acting as tumor suppressors or oncogenes. When they act as oncogenes, they usually downregulate the expression of tumor suppressor genes, leading to uncontrolled cell proliferation, inhibition of apoptosis, and other hallmarks of cancer. Although miRNA-200c is primarily known for its tumor-suppressive effects, under certain contexts, it can exhibit oncogenic properties [66]. For example, in ESCC, miRNA-200c is overexpressed and contributes to resistance against chemotherapy and radiotherapy [50]. Furthermore, elevated serum levels of miRNA-200c in ESCC patients are associated with an increased risk of mortality compared to those with lower expression levels [65]. This result is in line with a meta-analysis suggesting miRNA-200c as a (negative) prognostic marker for various solid malignancies [51,52]. Reducing miRNA expression through the suppression of EZH2 has been shown to lower the levels of EMT markers, such as N-cadherin, ZEB2, and Vimentin. Therefore, inhibiting miRNA-200c expression via EZH2 may significantly mitigate EMT in ESCC [53].

## Exosomes as vehicles for miRNA delivery in cancer treatment

### Extracellular vehicles (EVs)

Extracellular vehicles (EVs) are diverse lipid-bilayer particles released from various cell types, found in many biological fluids. They are categorized by size into exosomes (30–150 nm), ectosomes or microvesicles (100–1000 nm), apoptotic bodies (1–5 μm), and large oncosomes (1–10 μm). Exosomes form via endocytosis of the plasma membrane, creating early endosomes that develop into late endosomes and then multivesicular bodies (MVBs) [67]. MVBs contain intraluminal vesicles (ILVs) and can encapsulate cargo from cellular organelles, such as the trans-Golgi network (TGN) and endoplasmic reticulum (ER). Furthermore, MVBs are translocated to the membrane and, upon fusion, release vesicles into the extracellular space [68].

In the extracellular space, vesicles categorized as small (<200 nm) and medium/large (>200 nm) are collectively referred to as "extracellular vesicles." The membranes of these EVs are composed of a diverse array of lipids and proteins. In addition to proteins embedded within the membrane, EVs may also harbor biomolecules adsorbed on their surface, which are termed protein coronas [69]. EVs are released by cells and transport a range of biologically active molecules. These include lipids, RNA species, DNA, soluble proteins (such as enzymes, cytokines, chemokines, and growth factors), as well as various other proteins like tumor suppressors, oncoproteins, and regulators of transcription and splicing [70]. EVs can also transport a range of regulatory proteins, including those involved in extracellular matrix (ECM) remodeling [68], and intercellular signal transduction [69]. Due to their multiple cargoes, EVs are crucial in modulating the tumor microenvironment (TME) and can influence various aspects of cancer biology, including tumor development, immune evasion, angiogenesis, invasion, and metastasis [71]. In recent years, EVs have gained prominence as significant cancer biomarkers due to their elevated concentration in blood and other bodily fluids, as well as the biological information they convey from their cells of origin, including genetic and proteomic data. Interestingly, cancer cells release a greater quantity of EVs compared to normal cells, resulting in a higher concentration of these vesicles in the bloodstream of cancer patients relative to healthy individuals [72].

### Exosomes as a novel delivery vehicle for miRNAs in cancer therapy

The clinical application of miRNAs holds great promise, particularly in the areas of cancer therapy, diagnostics, and personalized medicine. However, several challenges must be addressed to fully realize their potential. For example, miRNAs are prone to rapid degradation in the bloodstream, which poses a significant hurdle for effective delivery to target tissues [73]. Furthermore, precise delivery of miRNAs to specific cells or tissues without affecting non-target cells is challenging. Non-specific delivery can lead to off-target effects, which may result in unintended consequences. Additionally, the introduction of synthetic miRNAs can trigger an immune response, leading to inflammation or other immune-related complications, which could limit their safety and efficacy [68].

Advances in nanotechnology and the use of exosomes as delivery vehicles are promising avenues to enhance the stability, targeting specificity, and efficiency of miRNA-based therapies. Small extracellular vehicles (EVs), namely exosomes present a compelling alternative to liposomal and polymeric drug delivery systems. Liposomal and polymeric nanoparticles (NPs) are used for targeted delivery of diverse pharmacological agents, including anticancer therapeutics and analgesics. Nonetheless, these NPs face certain constraints, such as susceptibility to variations in shear stress, thermal fluctuations, pH changes, and alterations in diluent concentration [74]. Additionally, they exhibit limitations in achieving precise delivery to specific cellular targets within the organism [71]. Here, exosomes represent an advantageous alternative due to their extended circulation time, excellent biocompatibility, minimal inherent toxicity, and capacity for targeted tissue delivery [75]

The unique structure of exosomes with their bilayer membranes facilitates the transfer of non-coding RNAs (ncRNAs) protecting them from degradation by circulating nucleases. The predominant types of ncRNAs found in exosomes include miRNA, long non-coding RNA (lncRNA), and circular RNA (circRNA) [76]. For example, exosomes derived from normal tongue epithelial cells overexpressing miRNA-200c can transfer the miRNA-200c to paclitaxel (PTX)-resistant tongue squamous cell carcinoma (TSCC) cells in vitro, thereby enhancing their sensitivity to PTX treatment [25]. Also in vivo, administration of the miRNA-200c-overexpressing exosomes significantly inhibited TSCC (tongue squamous cell carcinoma) growth in response to PTX therapy. The reduction in PTX resistance in the TSCC cells was primarily attributed to the targeting of TUBB3 and PPP2R1B by miRNA-200c. These findings suggest that exosome-mediated delivery of miRNA-200c represents a promising and effective strategy for overcoming chemical resistance in TSCC [69]. However, regarding the heterogeneity of HNSCC more basic and clinical research is urgently needed to prove miRNA-200c-mediated effects on chemoresistance, and thus patient outcome [2,3].

## Challenges and prospects in using exosomes as drug carriers

**Fig. 6:**
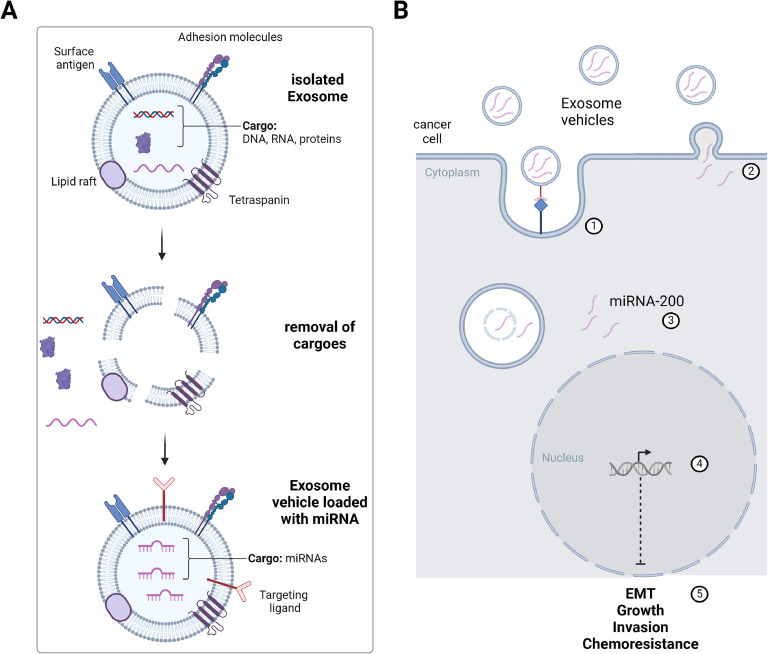
Application of engineered exosomes as drug carriers. A. The removal procedure of natural contents in cell-derived exosomes to construct exosome vehicles. Native, isolated exosomes contain endogenous cargoes, such as DNA, RNA, and proteins. The lipid layer of exosomes is disintegrated to remove endogenous cargoes, e.g. by hypotonic treatment. Then, exosomes can be re-formed and loaded with therapeutic molecules, here miRNA, e.g. by electroporation or sonication. Here, the surface of exosome vehicles can be modified with targeting ligands to increase delivery specificity. B. Uptake and possible effects of engineered exosomes containing miRNA-200. Uptake of exosome vehicles by cancer cells can occur via receptor-ligand-mediated endocytosis ①, and/or membrane fusion ②. Release of miRNA-200 cargo in the cytoplasm ③, and effects on transcriptional regulation ④ can inhibit epithelial-mesenchymal transition (EMT), reduce tumor cell growth and invasion, as wells as chemoresistance [5]. Created with BioRender.com.

Several studies indicated that exosome-mediated delivery of tumor-suppressive miRNAs, such as miRNA-200c, represents a promising approach to improve cancer patient treatment. However, there are also concerns about the use of exosomes as therapeutic vehicles [67,68,69]. In addition to cellular heterogeneity, exosomes can vary in size, content, and functional properties, which brings up concerns about their safety. Prior to advancing to clinical applications, it is crucial to first eliminate the endogenous contents of exosomes to mitigate potential pro-cancer effects. Subsequently, these exosomes should be engineered by incorporating targeting moieties and/or loading them with anti-cancer agents to develop customized therapeutic exosomes [67] (Fig. 6).

Researchers demonstrated the feasibility of using hypotonic treatment to eliminate the contents of exosomes derived from macrophages [76]. Following this process, the modified exosomes, when loaded with anti-cancer drugs, were capable of exhibiting anti-cancer effects against breast cancer [75]. Additional studies are highly recommended to explore the application of hypotonic treatment for exosomes derived from various cell types. This approach could help address exosomal heterogeneity and reduce the safety risks associated with using exosomes as drug delivery systems [77].

Besides the development of these improved delivery systems for miRNAs, future research should also focus on personalized approaches by miRNA profiling to assess a patient´s personal miRNA expression pattern. Furthermore, the exploitation of combination therapies along with comprehensive safety and efficacy studies will be crucial in translating miRNA-based therapies from the lab to the clinic [78].

## Conclusions

Exploring the tumor-suppressive role of miRNA-200c in head and neck squamous cell carcinoma (HNSCC) reveals significant potential for therapeutic intervention, particularly through exosome-mediated delivery. miRNA-200c is known for its ability to inhibit epithelial-to-mesenchymal transition (EMT) by downregulating transcriptional repressors of E-cadherin, such as ZEB1 and ZEB2. In HNSCC, where metastasis and chemoresistance are major challenges, restoring or enhancing miRNA-200c expression could suppress tumor progression and enhance treatment sensitivity. However, tumors of HNSCC patients are often diagnosed in an advanced state with metastases already present. Here, further studies are urgently needed elucidating the effects of miRNA-200c treatment in patients with metastatic disease. Previous studies suggest that molecular mechanisms of miRNA-200c may prevent further malignant progression also in these patients, but experimental evidence for this is still lacking. Regarding the complex roles of miRNA-200c in cancer, acting as both a tumor suppressor and, in some contexts, as an oncogene, it is mandatory to further characterize its specific effects influenced by the tumor localization, the microenvironment, and the genetic background of the tumor cells.

The use of exosomes presents a promising strategy for miRNA-200c delivery. Exosome-mediated delivery could overcome the limitations of direct miRNA administration, such as degradation and poor cellular uptake, ensuring targeted and effective delivery of miRNA-200c to tumor cells. This approach not only underscores the therapeutic promise of miRNA-200c in managing HNSCC, but also opens avenues for developing innovative treatments that harness the power of precision medicine in cancer therapy.

## Funding

This research was funded by the Brigitte und Dr. Konstanze Wegener Stiftung (project #90).

## CRediT authorship contribution statement

**Mohamed S. Kishta:** Writing – review & editing, Writing – original draft, Visualization, Supervision, Conceptualization. **Aya Khamis:** Writing – review & editing. **Hafez AM:** Writing – original draft. **Abdelrahman H. Elshaar:** Writing – original draft. **Désirée Gül:** Writing – review & editing, Writing – original draft, Visualization, Supervision, Funding acquisition, Conceptualization.

## Declaration of competing interest

The authors declare that they have no known competing financial interests or personal relationships that could have appeared to influence the work reported in this paper.
